# Commentary: Tobacco consumption and body weight: Mendelian randomization across a range of exposure

**DOI:** 10.1093/ije/dyv033

**Published:** 2015-03-30

**Authors:** Frank Dudbridge

**Affiliations:** Department of Non-communicable Disease Epidemiology, London School of Hygiene and Tropical Medicine, Keppel Street, London WC1E 7HT, UK. E-mail: frank.dudbridge@lshtm.ac.uk

Tobacco consumption is consistently associated with reduced body weight, creating an incentive to initiate smoking and a disincentive to cease, although the health risks associated with the habit outweigh the benefits of reduced weight. Among smokers however, increasing consumption has been associated with increased body weight. To determine whether this contradiction reflects causal processes, Winsløw *et al.*^1^ have applied Mendelian randomization (MR) in testing the association of a genetic variant, rs1051730 in *CHRNA3*, with measures of body weight among 80 342 members of the Copenhagen General Population Study. Among smokers, each minor (T) allele carried was associated with an increase of about one cigarette per day, but with a decrease in several measures of body weight, in contrast to the observational results. These results, in line with other recent studies,^2–6^ suggest that increased tobacco consumption causes reduced body weight, as does smoking itself.

Here I remark on two aspects of this study that may recur in other MR studies of this type: the restriction of genetic effects to current smokers, and the change, in the observational data, from decreasing to increasing body weight as cigarette consumption increases.

The associations of rs1051730 with decreased body weight are present in current smokers, but not in former or never smokers, suggesting that the gene acts on body weight only through its effect on smoking. However, by stratifying on smoking status, the results are potentially prone to collider bias, whereby an association is induced between the gene and confounders of the exposure-outcome association, creating a non-causal association between the gene and the outcome. The question is whether smoking status should be considered as derived from the quantity consumed (one who smokes zero cigarettes per day is a non-smoker, others are smokers, Figure 1 arrow a), or as an exogenous variable whose value constrains the possible consumption (non-smokers must smoke no cigarettes per day, smokers must smoke a positive number, Figure 1 arrow b). Under the former, stratifying on smoking status would entail a collider bias, and an association would be seen between genotype and smoking status; but such an association was not observed by Winsløw *et al*., nor was any association between rs1051730 and known confounders when stratifying on smoking status. Thus the data support the view that smoking status is distinct (i.e. has distinct determinants) from tobacco consumption (Figure 1 arrow b). Indeed it seems reasonable that individuals would generally decide whether or not to smoke as a precursor to developing their usual consumption. Nevertheless, collider bias can only be completely ruled out if the whole sample is analysed, and here the genetic associations remained significant owing to the sufficiently high proportion of smokers among them.

**Figure 1.**
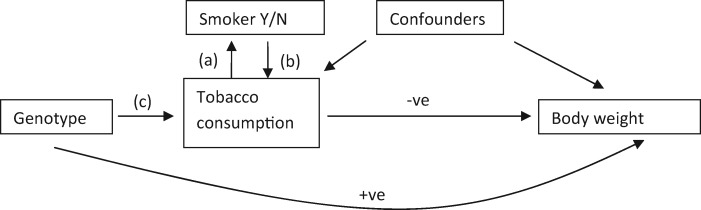
Directed acyclic graph showing causal relationships between genotype, smoking status and consumption, and body weight outcomes. Arrow (a) applies if smoking status is defined by the quantity consumed. Arrow (b) applies if smoking status has distinct determinants and defines the possible range of consumption. Arrow (c) is absent for non-smokers under (b). Y/N, yes or no; +ve, positive; -ve, negative.

The lack of genetic association among never smokers could be interpreted as evidence against alternative pathways to that through smoking, in line with MR assumptions. However, Taylor *et al*.^6^ reported positive association between rs1051730 and body mass index (BMI) (*P* = 6.4 × 10^−5^) in a sample of 66 809 never smokers. This trend is apparent in the 32 937 never smokers in Winsløw *et al*. (*P* = 0.07, their Figure 4) and would have reached significance (*P* = 0.01) if the same trend were observed in 66 809 subjects. Thus the exclusion restriction seems to be violated, but as the effect is in the opposite direction to that observed in smokers, it need not conflict with the qualitative conclusion that increased tobacco consumption reduces body weight.

Observational data show a decrease in body weight at low tobacco consumption, with increases in body weight at higher levels of consumption (Figure 2 of Winsløw *et al*.). This raises the question of whether the J-shaped pattern reflects causal effects: the marginal inverse association of rs1051730 may be driven by effects at certain consumption levels. Perhaps there is a particularly strong inverse genetic association at low consumption levels, which outweighs positive genetic associations at higher levels. The conclusion that increased smoking reduces weight might only be applicable at certain low levels of smoking.

Similar questions have recently been addressed in relation to alcohol consumption. rs1229984 in *ADH1B* is associated with reduced alcohol intake and also with reduced incidence of coronary heart disease (CHD) among drinkers, but is not associated with CHD among non-drinkers.^7^ This suggests beneficial effects of reduced intake, but observational data show a J-shape with increased risk in abstainers compared with moderate drinkers. Furthermore, for alcohol it is more plausible that abstention is a special case of low intake, perhaps due to unpleasant side effects associated with *ADH1B* variation. rs1229984 is indeed associated with drinking status,^7^ so stratification on drinking status is less defensible than it is for smoking status.

To address these questions, methods have recently been proposed to estimate localised causal effects that can indicate whether the direction of causal effect is constant over the range of exposure. A foundational result is that the marginal treatment effect is a weighted sum of local average treatment effects (LATEs), defined for subgroups of subjects with the same potential outcomes.^8^ Silverwood *et al*.^9^ considered the effect of alcohol intake on markers of cardiovascular disease. Using rs1229984 in *ADH1B,* they stratified subjects according to their potential alcohol intake if homozygous for the common allele. With some assumptions their approach explicitly calculates LATEs, and was used to infer J-shaped causal associations of alcohol intake on several cardiovascular markers including systolic blood pressure and BMI. Burgess *et al*.^10^ proposed a more general approach, also based on considering potential levels of exposure for a set genetic value. Because the ‘instrument-free exposure’ is by construction not associated with the genotype, stratification on it does not induce a collider bias. With arbitrary stratification, their approach estimates localized causal effects, distinct from formal LATEs but also able to identify non-linear patterns in the causal dose-response curve.

These approaches show promise for developing insight into the pattern of causal effects when observational data show variation in effect size or direction across the range of exposure. One limitation is that the localized effects apply to levels of the instrument-free exposure rather than the exposure itself, and this distorts their interpretation.^11^ Thus we can only make statements about subjects with given potential levels of exposure, rather than their actual exposures which are arguably more relevant. Another limitation is an assumption that the effect of genotype on exposure is constant for all subjects. This will not hold when there is a gene-environment interaction, as in the present case in which the gene only affects tobacco consumption in those who are smokers (Figure 1 arrow c); stratification on exposure levels may be required, with attendant possibilities of collider bias.

An increasing number of MR studies will consider dosage of exposures that are present among some subjects only. Such studies must carefully consider the relationship between presence and dosage of exposure, together with their genetic associations. When observational effects vary over the range of exposure, it will be informative to apply emerging methods for localized effects to infer non-linear causal effects. Such approaches will be particularly valuable when there are conflicts between causal and observational results, which merit exploring in greater detail.

## Funding

This work was funded by the MRC (K006215).

**Conflict of interest:** None declared.
